# 

**Published:** 1890-10-04

**Authors:** 


					UIIVV.v, W-
IB mm II H 1 and nurses placing an undue nutritious value on such preparations as Extract of: Meat, home- _ -
jfg mk mm toe patients wouldin <5?p ymP ^e&\eiloxx??itG? THROUGHOUT
I v IIIL Mr'S fft M jp m UHITED KIKGim
WITH EXTRA NURSING SUPPLEMENT
Imit'i
ot \ y ayrn. 210. Vol IX.] SattjbdAY,
Octobek 4th, 1890.
[One Pen'ny.

				

